# The association between serum uric acid and diabetic complications in patients with type 2 diabetes mellitus by gender: a cross-sectional study

**DOI:** 10.7717/peerj.10691

**Published:** 2021-01-13

**Authors:** Yimeng Hu, Qinge Li, Rui Min, Yingfeng Deng, Yancheng Xu, Ling Gao

**Affiliations:** 1 Department of Endocrinology & Metabolism, Renmin Hospital of Wuhan University, Wu Han, Hu Bei, China; 2 Department of Endocrinology, Zhongnan Hospital of Wuhan University, Wuhan, China; 3 Department of Internal Medicine, University of Texas Southwestern Medical Center, TX, USA

**Keywords:** Serum uric acid, Non-alcoholic fatty liver disease, Diabetic retinopathy, Diabetic nephropathy, Diabetic peripheral neuropathy

## Abstract

**Background:**

The relationship between serum uric acid (SUA) and several diabetic complications or co-morbidities remains a matter of debate. The study aims to explore the association between SUA levels and the prevalence of non-alcoholic fatty liver disease (NAFLD), diabetic retinopathy (DR), diabetic nephropathy (DN) and diabetic peripheral neuropathy (DPN) in patients with type 2 diabetes mellitus (T2DM).

**Methods:**

A total of 2,809 participants (1,784 males and 1,025 females) were included in this cross-sectional study. Clinical characteristics and the prevalence of each of the four diseases were analyzed based on gender-specific quartiles of SUA levels. The Pearson correlation analysis and linear-regression analysis were used to access the correlation between SUA levels and clinical characteristics. Furthermore, a binary logistic regression analysis was carried out to determine whether SUA was an independent risk factor for each of the four complications.

**Results:**

SUA levels were positively correlated to BMI, BUN, Scr and TG, but negatively associated with eGFR, HDL, FBG, 2h-PG and HbA1c% for the patients with T2DM. The prevalence of NAFLD and DN, but not DR or DPN, were increased with SUA levels from the first to the fourth quartile. Binary logistic regression further disclosed that SUA was an independent risk factor for NAFLD (ORs Male = 1.002, ^∗^*P* = 0.0013; ORs Female = 1.002, ^∗^*P* = 0.015) and DN (ORs Male = 1.006, ^∗^*P* < 0.001; ORs Female = 1.005, ^∗^*P* < 0.001), but not for DR and DPN. After adjustment for the confounders, SUA levels were significantly associated with NAFLD within the 3rd (ORs = 1.829, *P* = 0.004) and 4th quartile (ORs = 2.064, *P* = 0.001) for women, but not independently associated with SUA for man. On the other hand, our results revealed increased prevalence of DN for SUA quartile 2 (ORs = 3.643, *P* = 0.039), quartile 3 (ORs = 3.967, *P* = 0.024) and quartile 4 (ORs = 9.133, *P* < 0.001) in men; however, SUA quartiles were significantly associated with DN only for quartile 4 (ORs = 4.083, *P* = 0.042) in women

**Conclusion:**

For patients with T2DM, elevated SUA concentration is an independent risk factor for the prevalence of NAFLD and DN after adjustment for other indicators, but not DR or DPN.

## Introduction

Type 2 diabetes mellitus (T2DM), a metabolic disorder, is associated with various complications and co-morbidities that result in cardiovascular diseases, renal failure, blindness and neuropathy (Peterson & Hart, 2016). It is the long-term complications that account for the majority of mortality in patients with T2DM (IDF, 2019). It is therefore critical to identify a clinical biomarker which can be used for early detection and management of the development of diabetic complications and co-morbidities.

Uric acid is a product of purine degradation. Elevated uric acid causes a series of pathophysiological changes through inflammation, oxidative stress and activation of the renin-aldosterone-angiotensin system (RAAS), which subsequently promotes the initiation and progression of multiple diseases including metabolic syndrome (Xiong, Liu & Xu, 2019). Recently, serum uric acid (SUA) has been regarded as a risk factor for T2DM (Kodama et al., 2009) since hyperuricemia stimulates insulin secretion and aggravates insulin resistance (Hu et al., 2018; Zhong et al., 2019). A clinical study from 8,678 patients with gout reports that uric acid-lowering therapy improves insulin resistance and reduces the incidence of T2DM (Niu et al., 2018). However, a meta-analysis included six trials with 455 patients suggests that uric acid-lowering therapy with allopurinol may be effective at reducing glycemia, but such an improvement does not observed in patients with diabetes (Chen et al., 2020) , suggesting that the significance of uric acid to T2DM might not be restricted to the incidence. It is therefore suspected that elevated SUA has some impact on the development and progression of complications associated with T2DM. Indeed, the relationship between SUA and various diabetic complications remains a matter of debate. Whereas longitudinal analyses demonstrated a unidirectional relationship from hyperuricemia to NAFLD incidence and lowering SUA levels may prevent subsequent NAFLD during the follow-up in non-T2DM population (Ma et al., 2020), a cross-sectional study have disclosed that SUA is not independently associated with NAFLD in T2DM after rigorous adjustment for additional metabolic parameters (Xu et al., 2019). Therefore, the relationship between NAFLD and hyperuricemia is debatable. Similarly, multiple lines of epidemiologic evidences have supported a role of SUA in the development of diabetic retinopathy (DR) in both T1DM and T2DM (Bjornstad et al., 2014; Liang et al., 2016); however, some studies found that high SUA levels were not associated with increased risk of DR in patients with T2DM (Xia et al., 2020). Diabetic nephropathy (DN), another microvascular complication of T2DM, is becoming a dominant factor leading to chronic kidney diseases (CKD) and renal transplantation. Numerous clinical studies have suggested that SUA is not only an independent risk factor for early renal disease, but also a driving force for the progression of renal dysfunction. Bjornstad and colleagues reports that during a 5.7-years follow-up period, higher baseline SUA is independently associated with increased risk for elevated urinary albumin excretion and reduced estimated glomerular filtration rate (eGFR) in obese adolescents with T2DM (Bjornstad et al., 2019). However, a cohort study of 3895 individuals with T1DM in Finland shows that baseline SUA is not independently associated with progression of DN despite being independently associated with the decline in eGFR, suggesting that increased SUA level is merely an outcome of kidney damage (Ahola et al., 2017). In summary, it remains inconclusive whether SUA can be used as a diagnosis biomarker for diabetic complications and co-morbidities due to the controversy findings from animal and clinical studies.

Here we designed a cross-sectional study to examine whether SUA levels in T2DM are influenced by age, gender, body mass index (BMI), serum lipid, renal function and other characteristics. Meanwhile, we explored whether SUA is associated with the prevalence of diabetic complications or co-morbidities (including NAFLD, DR, DN and DPN) in T2DM with a goal to determine the independent risk factors for each of the four diseases. To illustrate the difference in the pathophysiology between males and females, all the aforementioned analyses were further conducted separately by gender.

## Materials and Methods

### Subjects

**Figure 1:**
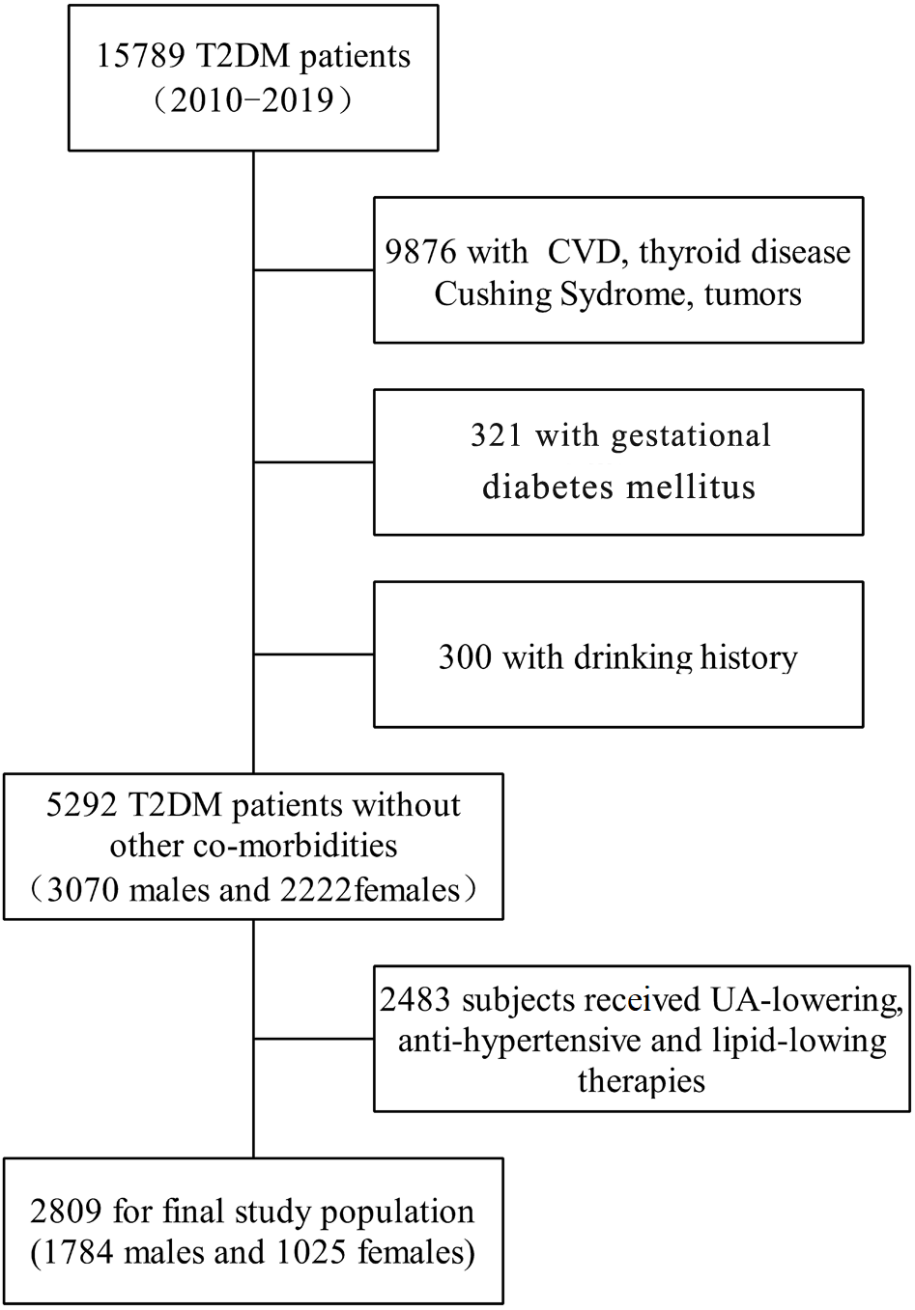
Flowchart of selection of study population.

The cross-sectional study enrolled patients with T2DM (20∼85 y), who were hospitalized in the Department of Endocrinology, Zhongnan Hospital of Wuhan University, China, between June 2010 and April 2019. Based on plasma glucose criteria, T2DM was diagnosed by fasting blood glucose (FBG) ≥ 7.0 mmol/L or 2 h plasma glucose (2 h PG) >11.1mmol/L during a 75 g oral glucose tolerance test (ADA, 2019). Patients with thyroid disease, Cushing syndrome, pheochromocytoma, malignant tumors, pregnancy, drinking history and those who were under uric acid-lowering, anti-hypertensive agents and lipid-lowering therapies were excluded. Finally, a total of 2,809 subjects (1,784 men and 1,025 women) were included in this retrospective study (Fig. 1). All the subjects received a low-sugar and low-fat diet and avoided excessive purine intake after their admission to the hospital. The study protocol has been approved by the Human Ethics Committee of Zhongnan Hospital, and all the participants had signed written informed consent (Ethical Application Ref: 2016019).

### Diagnose of diabetic complications or co-morbidities

#### Non-alcohol fatty liver disease (NAFLD)

Although pathologic examinations of liver biopsies are the gold standard for the diagnosis of NAFLD, it is impossible to obtain liver tissue from every participant. Therefore, the diagnosis criteria were as the following (Fan et al., 2011): (a). Intake of alcohol <140 g/week for males and 70 g/week for females in the past 12 months. (b). Results of liver ultrasonic examination. (c). Specific diseases that lead to steatosis can be excluded.

#### Diabetic retinopathy (DR)

DR is usually classified as non-proliferative diabetic retinopathy (NPDR) and proliferative diabetic retinopathy (PDR) with the former as the most common type of DR. The diagnosis of DR is by the lesions detected by ophthalmoscope examination which include microaneurysms, hemorrhages, soft exudates, hard exudates, neovascularization, and laser photocoagulation scars (Li et al., 2014). The examination was conducted at the Department of Ophthalmology and the images were interpreted by experienced and skilled ophthalmologists.

#### Diabetic nephropathy (DN)

Early diagnosis of DN can be done by screening for albuminuria, or glomerular filtration rate (GFR) (Komenda et al., 2014). DN is diagnosed when urinary albumin excretion is more than 30 mg per 24 h in consecutive sterile urine specimens or eGFR<60 mL/min per 1.73 m^2^ (Ahmad et al., 2019). The value of eGFR was calculated using the Chronic Kidney Disease Epidemiology Collaboration (CKD-EPI) equation (Levey et al., 2009). For males, eGFR = 141 × (Scr/0.9)−0.411 × (0.993)^age^ when Scr ≤ 0.9 mg/dL, while eGFR = 141 × (Scr/0.9)−1.209 × (0.993)^age^ when Scr >0.9 mg/dL. For females, when eGFR = 144 × (Scr/0.9)−0.329 × (0.993)^age^ when Scr ≤ 0.7 mg/dL, while eGFR = 141 × (Scr/0.9)−1.209 × (0.993)^age^ when Scr >0.7 mg/dL.

CKD is classified to 5 stages by eGFR categories (Hasegawa et al., 2019): stage 1 (eGFR ≥ 90), stage 2 (60<eGFR ≤ 89), stage 3 (30<eGFR ≤ 59), stage 4 (15<eGFR ≤ 29) and stage 5 (eGFR ≤ 15). When CKD has advanced to stage 3, the diagnosis of DN for T2DM patients will be confirmed.

#### Diabetic peripheral neuropathy (DPN)

DPN can be comprehensively evaluated with sensory and motor nerve conduction tests. Moreover, electrophysiological assessment has been used as an auxiliary examination to assess the conduction speed and latent-period of nerves. Clinically, the diagnosis of DPN includes the following aspects (Shabeeb et al., 2018): a. symptoms of nerve damage, including numbness, tingling, or pain in the extremities; b. physical examination, including muscle strength, reflexes, sensitivity to position changes, temperature, light touch and foot exam; c. report of macro electromyography that should be operated by neurological physician.

### Physical examination and laboratory measurements

All participants were required to take an interview to collect information about age, duration of T2DM, family disease history, alcohol consumption, smoking, and past medical conditions (Hu et al., 2018). When the patient was admitted to the hospital, a well-trained nurse would perform a simple physical examination, including height, weight, systolic blood pressure (SBP), diastolic blood pressure (DBP). BMI was calculated as weight in kilograms divided by the height in meters squared (kg/m^2^). In addition, blood samples were collected from patients’ antecubital vein at overnight fasting and 2 h after a 75 g oral glucose load. Total cholesterol (TC), triglycerides (TG), high-density lipoprotein-cholesterol (HDL-c), low-density lipoprotein-cholesterol (LDL-c), free fatty acid (FFA), blood urea nitrogen (BUN), serum creatinine (Scr), and serum uric acid (SUA), fasting blood glucose (FBG) and postprandial blood glucose (2h-PG) were measured on ADVIA^®^ 2400 Clinical Chemistry System (Siemens) according to the manufacturer’s instructions. HbA1c% was measured by enzymatic method using whole blood. To detect the levels of urinary albumin (Alb), patients were asked to collect urine for 24 h on the second day after admission.

### Statistical analysis

In this study, we used the multiple imputation method to impute the missing values by MI procedure in SAS 9.4 software (SAS Institute, Cary, NC). Sex-specific quartiles of SUA (µmol/L) were set up in male/female respectively: first quartile (Q1): <269.0/220.6 µmol/L, second quartile (Q2): 269.0–326.0/220.6–273.9 µmol/L, third quartile (Q3): 326.0–390.7/273.9–338.9 µmol/L, and fourth quartile (Q4): ≥ 390.7/338.9µmol/L. All continuous variables were presented as the mean ±  SD or median with interquartile range (IQR). One-way ANOVA was used to compare intergroup difference. Categorical variables were displayed as a number (proportion), and the intergroup comparison was performed by the chi-square test (*χ*2). The Pearson correlation analysis and linear-regression analysis were used to determine the correlation between SUA levels and age, duration of T2DM, BMI, SBP, DBP, TC, TG, HDL-c, LDL-c, FFA, BUN, Scr, eGFR, Alb, FBG, 2h-PG and HbA1c%. Binary logistic regression analysis was conducted to identify independent risk factors associated with the prevalence of the four diseases. We further performed logistical regression analysis using SUA quartile 1 as the reference group with adjustment for potential confounders to evaluate the specific association between the prevalence of diseases with SUA quartiles. The results were presented as odds ratios (ORs) and 95% confidence intervals (95% CIs). The results of multicollinearity test and goodness of fit test (Hosmer-Lemeshow) for each model were displayed in Table S1. A two-tailed *p* < 0.05 was considered statistically significant. All statistical analyses were conducted using SPSS software (version 21.0; SPSS, Chicago, IL, USA) and GraphPad Prism (version 8.0; GraphPad Software Inc., San Diego, CA, USA).

## Results

### Gender-specific clinical characteristics of participants according to SUA levels

Table 1 displayed the demographic and clinical characteristics of 2,809 participants including 1,784 men and 1,025 women in the four SUA quartiles, respectively. In males, BMI (*P* < 0.001), SBP (*P* = 0.001), BUN (*P* < 0.001), Scr (*P* < 0.001), TC (*P* = 0.006) and TG (*P* < 0.001) had a significantly increasing trend across the SUA quartiles. In contrast, eGFR (*P* < 0.001), HDL (*P* < 0.001), LDL (*P* = 0.021), FBG (*P* < 0.001), 2h-PG (*P* < 0.001) and Hb1Ac% (*P* < 0.001) were lower in the quartile 4 than quartile 1. Age (*P* = 0.067), duration of T2DM (*P* = 0.84) and DBP (*P* = 0.127), however, showed no statistical difference across the SUA quartiles in male. Different from the male, female participants showed no significant difference in SBP (*P* = 0.15), DBP (*P* = 0.48), TC(*P* = 0.63) and FFA (*P* = 0.22) across the four quartiles. Whereas age (*P* = 0.001), BMI (*P* = 0.001), BUN (*P* < 0.001), Scr (*P* < 0.001), Alb (*P* = 0.005) and TG (*P* < 0.001) showed a trend of increasing across the SUA quartiles in the female, eGFR (*P* < 0.001), HDL-c (*P* < 0.001), LDL-c (*P* = 0.009), FBG (*P* < 0.001), 2h-PG (*P* < 0.001) and HbA1c% (*P* < 0.001) were significantly decreased with ascending SUA quartiles

**Table 1:** Clinical characteristics of all participants according to quartiles of serum uric acid levels.

		Serum uric acid quartiles				
	Overall	Quartile 1	Quartile 2	Quartile 3	Quartile 4	P for trend
Male Characteristics	*n* = 1, 784	*n* = 445	*n* = 447	*n* = 446	*n* = 446	
serum uric acid range (µmol/L)		<269.0	269.0–326.0	326.0-390.7	>390.7	
age (year)	54.2 ± 12.1	55.5 ± 11.4	53.4 ± 11.2	54.0 ± 12.7	53.8 ± 12.8	0.067
duration (years)	5.0(1.0,10.0)	5(1.0,10.0)	5.0(1.0,10.0)	5.0(1.0,10.0)	5.0(1.0,10.0)	0.84
BMI(kg/m^2^)	25.3 ± 3.3	24.3 ± 3.3	25.3 ± 2.9	25.7 ± 3.3	26.3 ± 3.3	<0.001^*^
SBP(mmHg)	130.1 ± 17.2	128.0 ± 16.7	129.3 ± 17.6	131.6 ± 16.5	131.3 ± 17.8	0.001^*^
DBP(mmHg)	78.4 ± 10.8	77.6 ± 10.0	78.6 ± 11.2	78.3 ± 10.1	79.2 ± 11.7	0.127
BUN(mmol/L)	5.7 ± 1.6	5.4 ± 1.5	5.5 ± 1.4	5.8 ± 1.6	6 ± 1.9	<0.001^*^
Scr( µmol/L)	71.2(62.6,82.3)	65.7(58.8,74.0)	69(61.5,79.9)	72.2(64.2,83.0)	78.2(67.8,90.1)	<0.001^*^
eGFR(mL/min/1.73m^2^)	107.3(91.3,118.7)	110.1(99.4,119.8)	109.4(94.2,120.6)	107.2(89.4,118.7)	100.1(80.0,113.6)	<0.001^*^
A(mg/24h)	12.5(5.9,22.7)	11.8(5.7,22.7)	11.6(5.8,22.7)	13.7(6.5,22.7)	13.0(5.9,22.7)	0.089
TC(mmol/L)	4.7 ± 1.2	4.6 ± 1.0	4.8 ± 1.1	4.7 ± 1.1	4.8 ± 1.1	0.006^*^
TG(mmol/L)	1.8(1.2,2.9)	1.4(1.0,2.1)	1.7(1.2,2.9)	1.8(1.2,3.0)	2.2(1.4,3.7)	<0.001^*^
HDL-c(mmol/L)	0.99 ± 0.3	1.1 ± 0.3	1.0 ± 0.3	0.99 ± 0.3	0.93 ± 0.2	<0.001^*^
LDL-c(mmol/L)	2.8 ± 0.9	2.9 ± 0.8	2.9 ± 0.9	2.8 ± 0.9	2.7 ± 0.9	0.021^*^
FFA( µmol/L)	552.3(404.7,635.8)	508.6(391.8,612.1)	522.4(399.9,631.2)	535.5(407.0,649.7)	533.4(413.9,649.8)	0.106
FBG(mmol/L)	8.4(6.3,11.3)	9.3(6.8,12.1)	8.4(6.3,11.9)	8.0(6.2,10.9)	7.8(6.0,10.4)	<0.001^*^
PBG(mmol/L)	18.1 ± 5.4	19.4 ± 5.4	18.4 ± 5.3	17.7 ± 5.1	16.9 ± 5.3	<0.001^*^
HbA1c(%)	8.5(7.0,10.3)	9.3(7.6,11.1)	8.6(7.1,10.4)	8.3(7.0,9.9)	7.8(6.7,9.6)	<0.001^*^
Female Characteristics	*n* = 1, 025	*n* = 255	*n* = 258	*n* = 256	*n* = 256	
serum uric acid range (µmol/L)		<220.6	220.6-273.9	273.9-338.9	>390.7	
age (year)	60.1 ± 11.3	58.6 ± 10.7	59.6 ± 10.1	60.3 ± 10.9	61.8 ± 13.2	0.001^*^
duration (years)	6.0(2.0,10.0)	5.0(1.0,10.0)	6.0(1.0,10.0)	7.0(2.3,10.0)	7.0(2.0,13.0)	0.001^*^
BMI(kg/m^2^)	24.9 ± 3.6	24.0 ± 3.2	24.8 ± 3.9	25.1 ± 3.4	25.8 ± 3.8	<0.001^*^
SBP(mmHg)	131.3 ± 18.6	129.3 ± 18.1	132.4 ± 19.4	131.5 ± 19.4	132.1 ± 17.6	0.15
DBP(mmHg)	74.7 ± 10.6	74.7 ± 10.6	75.0 ± 10.5	75.4 ± 9.5	73.9 ± 11.5	0.48
BUN(mmol/L)	5.3 ± 1.8	4.9 ± 1.3	5.0 ± 1.4	5.3 ± 1.4	6.0 ± 2.6	<0.001^*^
Scr(µmol/L)	55.0(47.2,64.0)	50.4(44.1,56.9)	52.8(46.0,61.0)	56.3(49.0,65.2)	62.2(52.5,76)	<0.001^*^
eGFR(mL/min/1.73 m^2^)	97.5(87.4,107.5)	101.8(93.4,111.0)	99.4(91.7,108.6)	96.2(84.8,104.3)	90.1(70.0,102.5)	<0.001^*^
ALB(mg/24h)	11.7(5.7,21.8)	9.8(5.1,21.8)	11.0(5.1,21.8)	11.6(5.7,21.8)	14.8(6.6,22.1)	0.005^*^
TC(mmol/L)	4.8 ± 1.2	4.8 ± 1.1	4.8 ± 1.2	4.8 ± 1.3	4.7 ± 1.3	0.63
TG(mmol/L)	1.7(1.2,2.4)	1.4(1.0,1.4)	1.6(1.1,2.3)	1.9(1.3,2.8)	2.0(1.3,2.9)	<0.001^*^
HDL-c(mmol/L)	1.1 ± 0.3	1.2 ± 0.3	1.1 ± 0.3	1.1 ± 0.3	1.0 ± 0.3	<0.001^*^
LDL-c(mmol/L)	2.8 ± 1.0	3.0 ± 0.9	2.9 ± 1.0	2.9 ± 1.1	2.7 ± 1.0	0.009^*^
FFA( µmol/L)	522.5(400.7,628.6)	522.9(393.9,620.5)	504.2(387.6,642.8)	524.9(406.2,637.5)	540.3(416.9,630.1)	0.22
FBG(mmol/L)	8.1(6.0,10.9)	9.3(6.6,12.3)	8.4(5.7,11.3)	7.6(5.7,10.2)	7.9(6.2,9.7)	<0.001^*^
2h-PG(mmol/L)	18.4 ± 5.7	20.0 ± 5.7	18.6 ± 5.8	17.3 ± 5.3	17.6 ± 5.0	<0.001^*^
HbA1c(%)	8.3(7.0,10.1)	9.1(7.5,11.1)	8.2(6.7,10.3)	8.0(6.7,9.6)	7.8(6.9,9.4)	<0.001^*^

**Notes.**

SUA serum uric acid BMI body mass index SBP systolic blood pressure DBP diastolic blood pressure BUN blood urea nitrogen Scr serum creatinine eGFR estimated glomerular filtration rate ALB urinary microalbumin TC total cholesterol TG triglycerides HDL-c high-density lipoprotein-cholesterol LDL low-density lipoprotein-cholesterol FFA free fatty acid FPG fasting plasma glucose 2h-PG 2 h postprandial plasma glucose HbA1c% glycosylated hemoglobin

Data are expressed as mean ± SD, number (percentage), and median (interquartile ranges). *P* for trend was used to test the linear variation trend between clinical characteristic and SUA quartiles.

* Represented that the difference was significant.

### Correlations between SUA and clinical characteristics by gender

**Figure 2:**
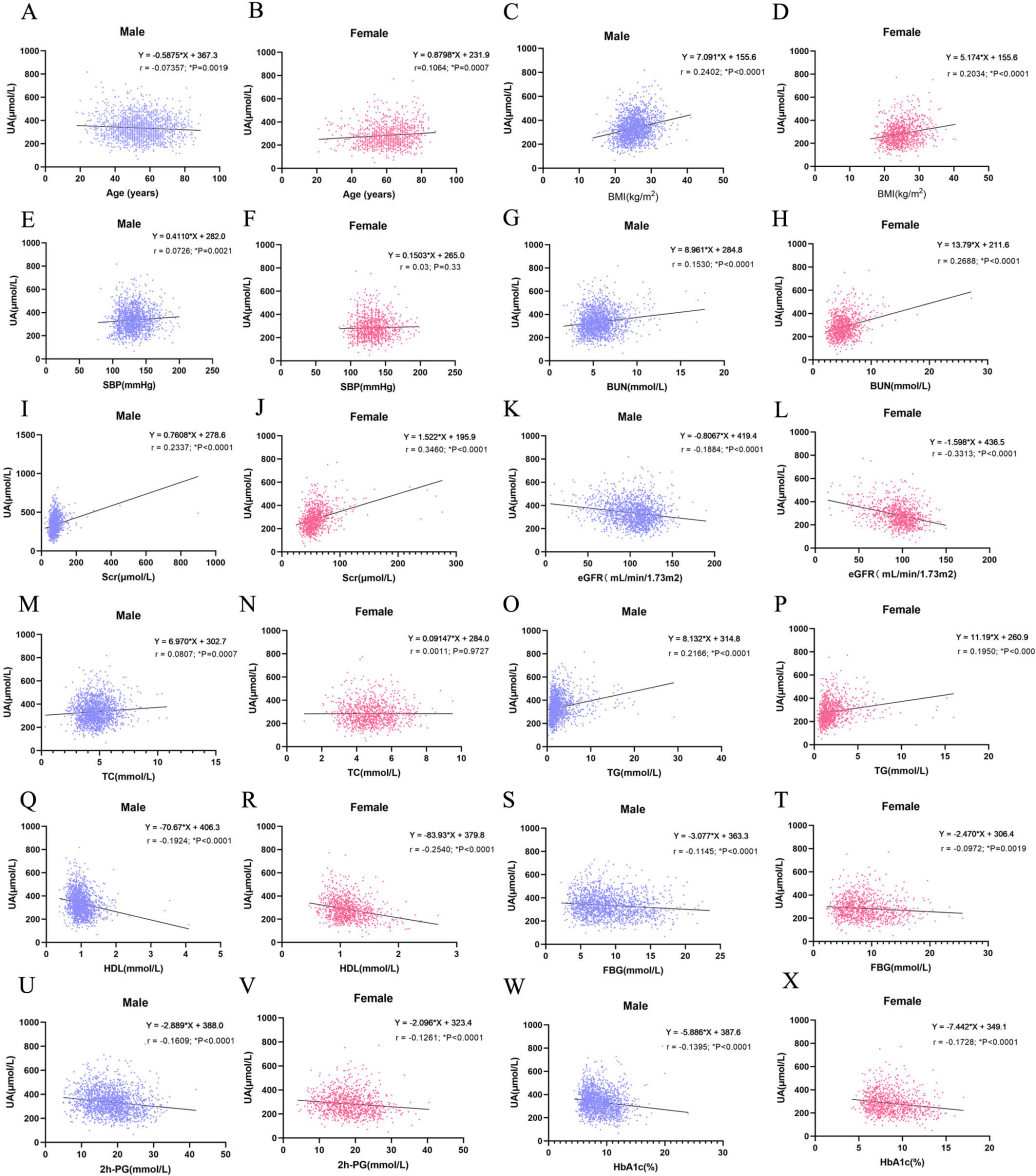
Correlation analysis between serum uric acid (SUA) with clinical characteristics by gender using Pearson correlation analysis and linear-regression analysis. (A, B) Correlation analysis between SUA and age in male (A) and female (B); (C, D) correlation analysis between SUA and BMI in male (A) and female; (E, F) correlation analysis between SUA and SBP in male (E, F) correlation analysis between SUA and SBP in male (E) and female (F); (E) and female (F); (I, J) correlation analysis between SUA and Scr in male (I) and female (J); (K, L) correlation analysis between SUA and eGFR in male (K) and female (L); (M, N) correlation analysis between SUA and TC in male (M) and female (N); (O, P) correlation analysis between SUA and TG in male (O) and female (P); (Q, R) correlation analysis between SUA and HDL-c in male (Q) and female (R); (S, T) correlation analysis between SUA and FBG in male (S) and female (T); (V, W) correlation analysis between SUA and 2h-PG in male (V) and female (W); (X, Y) correlation analysis between SUA and HbA1c% in male (X) and female (Y). r represents Pearson r value; an asterisk (*) represents *P* < 0.05 (two tailed).

As demonstrated in Fig. 2, SUA levels were positively correlated to BMI (*r* = 0.24, *P* < 0.0001; *r* = 0.20, *P* < 0.0001), BUN (*r* = 0.15, *P* < 0.0001; *r* = 0.27, *P* < 0.0001), Scr (*r* = 0.23, *P* < 0.0001; *r* = 0.35, *P* < 0.0001) and TG (*r* = 0.22, *P* < 0.000; *r* = 0.20, *P* < 0.0001) in male and female, respectively. Meanwhile, SUA levels were negatively correlated to eGFR (*r* =  − 0.18, *P* < 0.0001; *r* =  − 0.33, *P* < 0.0001), HDL (*r* =  − 0.19, *P* < 0.0001; *r* =  − 0.25, *P* < 0.0001), FBG (*r* =  − 0.11, *P* < 0.0001; *r* =  − 0.10, *P* = 0.002), 2h-PG (*r* =  − 0.16, *P* < 0.0001; *r* =  − 0.13, *P* < 0.0001), and HbA1c% (*r* = 0.-0.14, *P* < 0.000; *r* =  − 0.17, *P* < 0.0001) in male and female, respectively. The correlation of SUA with age, SBP, and TC showed some differences between men and women. In male, SUA showed a reducing tendency with incremental age in males ( *r* =  − 0.07, *P* = 0.02), while in female, SUA showed a positive association with age (*r* = 0.11, *P* = 0.0007). In male, SUA was positively correlated to SBP and TC (*r* = 0.07, *P* = 0.02; *r* = 0.08, *P* = 0.0007), while in female, no correlation was observed (*r* = 0.03, *P* = 0.33; *r* = 0.001, *P* = 0.97). In summary, HDL, BMI, BUN, Scr, eGFR and TG showed strong correlation with SUA, among which Scr seems to have the strongest impact on SUA for both genders.

### Association of SUA level with prevalence rate of diabetic complications

The prevalence of NAFLD was significantly different across the SUA quartiles in both men and women (Table 2). From the first to the fourth quartile, the prevalence rate was 34.60%, 43.60%, 46.90% and 48.70% in male, and 32.20%, 43.30%, 50.00% and 51.60% in female, respectively. The results indicated that the prevalence of NAFLD increased with the progression of hyperuricemia. Similar to NAFLD, the prevalence of DN was significantly different across the SUA quartiles in both men and women. From the first to the fourth quartile, the prevalence rate was 0.9%, 2.7%, 5.2% and 9.4%in male, and 1.2%, 0.8%, 3.9% and5.4% in female, respectively.

**Table 2:** The prevalence of diabetic complications across SUA quartiles in participants by gender.

	Serum uric acid quartiles				
	Quartile 1	Quartile 2	Quartile 3	Quartile 4	P for trend
Male	*n* = 445	*n* = 447	*n* = 446	*n* = 446	
NAFLD (%)	34.6	43.6	46.9	48.7	<0.05^*^
Diabetic retinopathy (%)	7.9	8.5	9	6.3	0.451
Diabetic peripheral neuropathy (%)	10.1	11.6	12.1	12.6	0.254
Diabetic nephropathy (%)	0.9	2.7	5.2	9.4	<0.05^*^
CKD1 (%)	90.6	84.8	81.4	73.8	<0.001^*^
CKD2 (%)	8.5	12.85	13.9	17	<0.001^*^
CKD3 (%)	0.7	2.5	4.7	8.3	<0.001^*^
CKD4 (%)	0	0.2	0.2	0.45	0.241
CKD5 (%)	0	0	0	0.45	0.651
Female	*n* = 255	*n* = 258	*n* = 256	*n* = 256	
NAFLD (%)	33.3	43.4	50	52	<0.001^*^
Diabetic retinopathy (%)	1.2	0.8	3.9	5.4	0.168
Diabetic peripheral neuropathy (%)	9	8.6	12.1	9.1	0.525
Diabetic nephropathy (%)	11.8	11.3	11.7	12.6	<0.01^*^
CKD1 (%)	87.1	86.7	78.1	66.5	<0.001^*^
CKD2 (%)	10.6	10.2	17.6	20.5	<0.002^*^
CKD3 (%)	0.8	0.8	1.6	9.4	<0.003^*^
CKD4 (%)	0	0	0.4	1.2	0.22
CKD5 (%)	0	0	0	0	/

**Notes.**

*P* for trend was used to test the linear variation trend between each of the complications and SUA quartiles.

* The difference was significant.

Based on eGFR value, we performed CKD grading for all the participants (Table 2) and found that the majority of T2DM patients were in CKD1 stage, suggesting that the deterioration of renal function is delayed in the current cohort which might be a result from glucose-lowing treatment. The proportion of CKD1was decreased with SUA in both genders. From the first to the fourth SUA quartile, the proportion was 90.6%, 84.85, 81.4%, and 73.8% in male, and 87.1%, 86.7%, 78.1% and 66.5% in female, respectively. In contrast, the proportions of CKD2 and CKD3 were higher in the fourth quartile than the first quartile.

Different from NAFLD and DN, the prevalence rate of DR ( *P* = 0.45) and DPN (*P* = 0.25) had no significant differences among the four groups for men and women. Taken together, our findings indicate that the association of SUA quartiles and the diabetic complications was similar in men and women.

### Identification of independent risk factors for the four diabetic complications

To identify independent risk factors for the four diseases associated with T2DM, binary logistic regression analysis was performed for each one. The results for males and females were displayed in Tables 3 and 4.

**Table 3:** Binary logistic regression analysis on related variables for the prevalence of diabetic complications in men.

	**Odds Ratio**	**95% CI**	*P* value
	**NAFLD**		
BMI	1.196	1.154–1.239	<0.001
TG	1.145	1.055–1.242	0.001
SUA	1.002	1.000–1.003	0.001
DBP	1.015	1.003–1.027	0.018
	**Diabetic retinopathy**		
Duration	1.062	1.029–1.096	<0.001
FFA	0.999	0.998–1.000	0.012
BMI	1.067	1.008–1.131	0.026
LDL	0.583	0.353–0.963	0.035
	**Diabetic nephropathy**		
Age	1.098	1.065–1.132	<0.001
BUN	2.146	1.816–2.536	<0.001
SUA	1.006	1.003–1.009	<0.001
SBP	1.021	1.005–1.038	0.01
	**Diabetic peripheral neuropathy**		
FBG	1.124	1.046–1.208	0.0015
Duration	1.041	1.015–1.068	0.002
HDL	2.163	1.192–3.924	0.011

**Notes.**

SUA serum uric acid BMI body mass index SBP systolic blood pressure BUN blood urea nitrogen HDL-c high-density lipoprotein-cholesterol FPG fasting plasma glucose

Participants without non-alcoholic fatty liver disease (NAFLD) / diabetic retinopathy (DR)/ diabetic nephropathy (DN) /diabetic peripheral neuropathy (DPN) were defined as 0 and those with NAFLD/ DR/DN/DPN as 1. *P*-value derived binary logistic regression analysis. Only the variables whose *p* < 0.05 were shown in the table.

**Table 4:** Binary logistic regression analysis on related variables for the prevalence of diabetic complications in women.

	**Odds Ratio**	**95% CI**	*P* value
	**NAFLD**		
BMI	1.192	1.43–1.244	<0.001
TG	1.436	1.226,1.682)	<0.001
HDL	0.486	0.274–0.861	0.014
SUA	1.003	1.001–1.004	0.005
	**Diabetic retinopathy**		
Duration	1.045	1.011-1.079	0.010
	**Diabetic nephropathy**		
Age	1.101	1.051–1.153	<0.001
BUN	2.103	1.715–2.579	<0.001
SUA	1.005	1.002–1.008	0.00
	**Diabetic peripheral neruopathy**		
Duration	1.067	1.035–1.099	<0.001

**Notes.**

SUA serum uric acid BMI body mass index SBP systolic blood pressure BUN blood urea nitrogen TG triglycerides HDL-c high-density lipoprotein-cholesterol LDL low-density lipoprotein-cholesterol

*P*-value derived Binary logistic regression analysis. Only the variables whose *p* < 0.05 were shown in the table.

For NAFLD, BMI (ORs = 1.196, *P* < 0.001; ORs = 1.201, *P* < 0.001), TG (ORs = 1.145, *P* = 0.001; ORs = 1.370, *P* < 0.001) and SUA (ORs = 1.002, *P* = 0.001; ORs = 1.002, *P* = 0.015) were the independent risk factors for both men and women. Besides, DBP (ORs = 1.015, *P* = 0.018) was a risk factor only for men, while LDL (ORs = 1.686, *P* = 0.005) was a risk factor only for women.

For DR, duration of T2DM was the common risk factor for both genders (ORs_Male_ = 1.062, *P* < 0.001; ORs_Female_ = 1.045, *P* = 0.006).In males, BMI (ORs = 1.067, *P* = 0.026) was a risk factor , whereas LDL (ORs = 0.583, *P* = 0.035) and FFA (ORs = 0.999, *P* = 0.012) were protect factors.

For DN, age (ORs = 1.098, *P* < 0.001; ORs = 1.101, *P* < 0.001), BUN (ORs = 2.146, *P* < 0.001; ORs = 2.103, *P* < 0.001), SBP (ORs = 1.021, *P* = 0.01; ORs = 1.022, *P* = 0.042) and SUA (ORs = 1.006, *P* < 0.001; ORs = 1.005, *P* = 0.005) were found as positive impact factors for men and women.

For DPN, only duration of T2DM (ORs_Male_ = 1.041, *P* = 0.002; ORs_Female_ = 1.067, *P* < 0.001) was a risk factor for both genders. Furthermore, FBG (ORs = 1.124, *P* = 0.002) and HDL (ORs = 2.163, *P* < 0.001) were independently associated with the prevalence of DPN in males.

The results of regression analysis suggested that SUA plays a role in promoting the prevalence of NAFLD and DN for patients with T2DM, however, the effect was not strong. Therefore, logistic regression analysis was performed using SUA quartile 1 as the reference group to explore further the specific effect of SUA on NAFLD and DN. As shown in Table 5, the crude ORs of NAFLD (95% CI) were 1.462 (1.116, 1.916), 1.666 (1.272, 2.182), and 1.791 (1.368, 2.344) from the second to the fourth quartile of SUA for male, and 1.618 (1.129, 2.320), 2.110 (1.473, 3.022), and 2.281 (1.593, 3.268) for female, respectively. Based on the adjusted model, the ORs of NAFLD (95% CI) were 1.260 (0.942, 1.684), 1.347 (0.999, 1.814) and 1.292 (0.941, 1.774) from the Q2 to the Q4 SUA for males, however, the difference was no longer statistically significant. Different from male, the ORs after adjustment in female were 1.829 (1.217, 2.749) and 2.064 (1.331, 3.203) in the Q3and Q4 SUA, indicating that the effect of SUA on the prevalence of NAFLD in female was stronger than male.

**Table 5:** Logistic regression analysis of the association between quartiles of SUA and the prevalence of NAFLD and DN with T2DM.

	**Male**			**Female**		
	N	ORs (95% CI)	*P* value	N	ORs (95% CI)	*P* value
**NAFLD**						
**Model 1**						
Q1	455	–	–	255	–	–
Q2	447	1.462 (1.116, 1.916)	0.006^*^	258	1.618 (1.129, 2.320)	0.006^*^
Q3	446	1.666 (1.272, 2.182)	<0.001^*^	256	2.110 (1.473, 3.022)	<0.001^*^
Q4	446	1.791 (1.368, 2.344)	<0.010^*^	256	2.281 (1.593, 3.268)	<0.001^*^
**Model 2**						
Q1	455	–	–	255	–	–
Q2	447	1.260 (0.942, 1.684)	0.106	258	1.467 (0.986, 2.182)	0.059
Q3	446	1.347 (0.999, 1.814)	0.051	256	1.829 (1.217, 2.749)	0.004
Q4	446	1.292 (0.941, 1.774)	0.113	256	2.064 (1.331, 3.203)	0.001^*^
**DN**	N	ORs (95%CI)	*P* value	N	ORs (95%CI)	*P* value
7l**Model 1**
Q1	455	–	–	255	–	–
Q2	447	3.041 (0.973, 9.503)	0.056	258	2.000 (0.495, 8.085)	0.331
Q3	446	5.995 (2.056,17.479)	0.001^*^	256	3.415 (0.929, 12.556)	0.065
Q4	446	11.462 (4.074, 32.242)	<0.001^*^	256	16.486 (2.039,53.941)	<0.001^*^
**Model 2**						
Q1	455	–	–	255	–	–
Q2	447	3.643 (1.067,12.439)	0.039^*^	258	1.516 (0.332, 6.928)	0.592
Q3	446	3.967 (1.203, 13.083)	0.024^*^	256	2.095 (0.426, 7.456)	0.426
Q4	446	9.133 (2.839, 29.373)	<0.001^*^	256	4.083 (1.035, 16.109)	0.042^*^

**Notes.**

Quartiles based on serum uric acid levels: first quartile, <269 µmol/L (<P25); second quartile, 269-326 µmol/L (P25-P50); third quartile, 326–390.7 µmol/L (P50-P75); and fourth quartile,>390.7µmol/L (>P75) (Males). Quartiles based on serum uric acid levels: first quartile, <220.6 µmol/L (<P25); second quartile, 220.6–273.9µmol/L (P25-P50); third quartile, 273.9–338.9 µmol/L (P50-P75); and fourth quartile, >338.9 µmol/L(>P75) (Females).

Data are presented as odds ratio (95% confidence interval) compared with first quartile 1. Participants without non-alcoholic fatty liver disease (NAFLD) / diabetic nephropathy (DN) were defined as 0 and those with NAFLD/ DN as 1. Model 1 for NAFLD: unadjusted variables; Model 2 for NAFLD: adjusted for age, BMI, duration, SBP, DBP, BUN, Scr, Alb, eGFR, TC, TG, HDL-c, LDL-c, FFA, FBG, PBG and HbA1c. Model 1 for DN: unadjusted variables; Model 2 for DN: adjusted for age, BMI, duration, SBP, DBP, BUN, TC, TG, HDL-c, LDL-c, FFA, FBG, PBG and HbA1c.

* The difference was significant.

The crude ORs of DN (95% CI) were 3.041 (0.973, 9.503), 5.995 (2.056, 17.479), and 11.462 (4.074, 32.242) in male, and 2.0 (0.495, 8.085), 3.415 (0.929, 12.456), and 2.447 (1.478, 4.049) in female, respectively. Based on the adjusted model, the ORs of DN from Q2 to the Q4 SUA were 3.643 (1.067,12.439), 3.967 (1.203, 13.083) and 9.133 (2.839, 29.373) in men, which were statistically significant. Nevertheless, the ORs of DN were significantly increased only for the Q4 SUA in women [4.083 (1.035, 16.109)], which implied the impact of SUA on the prevalence of DN in male was more powerful than female.

## Discussion

The current population-based study of T2DM presented data stratified according to SUA quartiles in a sex-specific manner. The analysis found that SUA levels were positively correlated with BMI, BUN, Scr and TG, and negatively correlated with eGFR, HDL, FBG, 2h-PG and HbA1c% in both genders. Given the fact that BUN, Scr, and eGFR are the parameters reflecting the ability of glomeruli clearance, it can be inferred that the elevation of SUA level in T2DM is mainly attributed to the decline of the kidney function in terms of UA clearance. However, it also suggested that hyperuricemia impaired glomerular filtration, which subsequently led to the changes of BUN, Scr, and eGFR levels. Nevertheless, a causal relationship of SUA and the development of hypertension has been shown regardless of baseline renal function (Turak et al., 2013), which suggests that a role of SUA on blood pressure is unrelated to renal clearance. In addition, SUA levels are increased with BMI, congruent with the findings that obesity might cause overproduction and underexcretion of UA (Matsuura et al., 1998). Moreover, it has been reported that lowering SUA by benzbromarone significantly attenuates FFA elevation and promotes adipose tissue beiging via AMPK activation, and subsequently reduces adiposity in mice, suggesting that increased UA contribute to the development of metabolic abnormalities in adipose tissue (Su et al., 2020b). It didn’t make sense that SUA was negatively corelated with FBG, 2h-PG and HbA1c% which reflected blood glucose levels, which was consistent with previous study (Xu et al., 2019). However, a meta-analysis of 12 cohort studies reveals a positive nonlinear relationship between SUA levels and impaired fasting glucose (Jia et al., 2013). One of the possible reasons for this phenomenon is that hyperuricemia could promote insulin secretion to compensate for insulin resistance in the short term (Hu et al., 2018). In addition, we couldn’t eliminate the effect of hypoglycemic agent in this study, which might result in the relationship. Interestingly, the positive correlation between SUA and SBP was only observed in males but not females in the current study. Patients with hypertension often suffer from hyperuricemia due to the obstruction of UA excretion, however, the incidence of cardiovascular events stills soared 3-5 times even when their blood pressure is under strict control (Franse et al., 2000). This observation indicates that hyperuricemia can be an independent risk factor of cardiovascular events rather than a mere result of hypertension and renal impairment. However, a longitudinal study based on 1,666 T2DM patients concludes that SUA is not related to the incidence of hypertension (Janghorbani et al., 2018), suggesting an ambiguous role of UA in those diseases.

In this study, the prevalence of NAFLD in patients with T2DM was found increased from the first to the fourth quartile of SUA. Many clinical researches have reported that elevated SUA levels are associated with higher prevalence and the severity of NAFLD (Darmawan, Hamijoyo & Hasan, 2017). However, very few studies have been conducted to evaluate the significance of SUA in NAFLD in patients with T2DM. The logistical regression models with rigorous adjustments for other risk factors indicate that SUA is a significant risk factor for NAFLD in female, but not in male. Notably, a similar finding has been reported in the non-diabetic population (Wu et al., 2015). Although the reason for this gender difference is still uncertain, sex hormones may play a role, especially that estrogen facilitates excretion of UA during the reproductive period (Borges et al., 2010; Lee, Kim & Kim, 2017) . With respect to the mechanism for SUA-mediated pathophysiology of NAFLD, UA has been reported to induce hepatic steatosis by stimulating fat acid synthesis, which precipitates the generation of mitochondrial oxidative stress, endoplasmic reticulum stress and SREBP-1c excessive activation (Choi et al., 2014; Lanaspa et al., 2012b). On the other hand, UA activates oxidative stress-induced lipid peroxidation, DNA damage and inflammatory response, which eventually generates cellular damage (Yu et al., 2010). Hepatocytes that exposed to uric acid for the long term are inclined to develop mitochondrial dysfunction and increase de novo lipogenesis, which jointly contributes to the incidence of NAFLD (Lanaspa et al., 2012b). It is well known that activity of the AMP-activated kinase (AMPK) is reduced in hepatic steatosis. UA, can inhibit AMPK activity in human hepatocytes (Lanaspa et al., 2012a)

Mounting studies have demonstrated that elevated SUA is an independent risk factor for the incidence of DN (Hayashino et al., 2016; Zoppini et al., 2012). Our studies found a significant increase in DN prevalence across SUA quartiles among subjects, especially in men, which highlighted a significant role of SUA on the development of DN in patients with T2DM. In order to evaluate the role of SUA as a risk factor for end stage renal disease (ESRD), the distribution of CKD was compared across SUA quartiles. The proportion of CKD1 was decreased with SUA, and the proportion of CKD2 and CKD3 were increased with the elevation in SUA, suggesting that SUA was a risk factor for kidney failure. Indeed, a cohort study that followed 2,367 patients with T2DM for a mean of 4.6 years found that SUA >  6.3 mg/dL (374.85 µmol/L) was an independent risk factor for the progression of CKD (Chang et al., 2016). The SUA value used in their study is comparable to the threshold value used for DN diagnosis here (SUA_Male_ >  390.7 and SUA_Female_ >  338.9 µmol/L). Interestingly, in moderate non-diabetic CKD, SUA ≥ 9 mg/dl is associated with higher mortality. However, once progressing to severe non-DM CKD, SUA <  5 mg/dl is associated with higher mortality, which suggested extremely reduced SUA means malnutrition (Lee & Tsai, 2020).

It has been reported that UA can damage microvascular, a mechanism for the pathogenesis of DN. Specifically, hyperuricemia leads to reduced production of endogenous nitric oxide (NO) and the injury of endothelial cells, which increases the oxidative stress and inflammation at glomerulus (Rock, Kataoka & Lai, 2013). Evidence from animal and clinical studies showed that UA-mediated RAS activation also is s a novel mechanism of UA-induced endothelial dysfunction, which is closely related to cardiovascular and kidney diseases, such as atherosclerosis or DN (Su et al., 2020a). Moreover, UA sodium deposited in the renal interstitium impairs renal tubular function. However, a recent double-blind trial failed to show the clinical benefits of serum urate reduction with allopurinol on kidney outcomes among patients with T1DM (Doria et al., 2020). The discrepancy in conclusions between this study with previous studies may be caused by the indirect predictive effect of SUA on kidney dysfunction, which can be ascribed to the association of SUA with other risk factors that are causally related to DN, such as insulin resistance (Wang et al., 2019).

In contrast to DN, neither DR nor DPN was found associated with SUA levels, which was consistent with the previous reports (Xia et al., 2020). On the contrary, a multicenter cross-sectional study in Thailand concludes that SUA is independently associated with peripheral neuropathy in T2DM patients, and elevated SUA should be considered a risk factor for DPN in clinical practice (Kaewput et al., 2020).Two explanations are suggested for the controversial findings. Firstly, the prevalence of DR and DPN were less than 12% in the current sectional-study, far below the average levels which are 20.8% and 20% for DR and DPN respectively in Asian people (IDF, 2019). This phenomenon might be caused by relatively increased awareness of T2DM in China, so the most of in-hospital patients with less complicated conditions. Secondly, the sample size of this study is limited to obtain a positive result. Nevertheless, the values of eGFR in the DR and DPN groups were lower than non-DR and non-DPN groups in this study (Supplemental Tables), which was in agreement with the findings by Yamamoto M et al. They have reported that simultaneous assessment of proteinuria and eGFR is necessary for appropriate evaluation of risks of severe eye complications in patients with diabetes (Yamamoto et al., 2019). Potential mechanisms for the development of DR in accordance with renal dysfunction may lie in the microvascular damage induced by inflammation, vascular endothelial dysfunction, and advanced glycation end products (Sabanayagam et al., 2014).

There are several limitations in this study. Firstly, this cross-section study lacks the power to track the alterations of SUA levels longitudinally during the progression of the diseases, making it difficult to determine the causality of SUA for diabetic complications. Secondly, the prevalence of diabetic complications was relatively lower than the average incidence in Asia, which could have hindered the discovery of significant difference among groups. Lastly, several antidiabetic drugs, such as metformin and SGLT-2 inhibitors could have profound effects on uric acid homeostasis, which could lead to altered SUA levels. Nevertheless, this population-based analysis reveals a significant link of SUA and a spectrum of clinical characteristics associated with T2DM. What’s more, this study comprehensively accesses the association between SUA levels and each of the four diabetic complications in a gender-dependent manner, which highlights the importance of clinical monitoring, early detection and sex-specific intervention for SUA to prevent the development of diabetic complications and co-morbidities.

## Conclusions

In summary, a positive correlation between SUA and prevalence of NAFLD/DN is observed in T2DM patients for both genders. The correlation is subjected to the influence of several confounders, such that after adjustment elevated SUA is still an independent risk factor for the prevalence of NAFLD for female when the SUA values fall in the Q3 and Q4. Although SUA quartiles are positively associated with the prevalence of DN for males, the association exists only when the SUA values fall in theQ4 for females.

##  Supplemental Information

10.7717/peerj.10691/supp-1 Supplemental Information 1 Multicollinearity test and goodness of fit test

10.7717/peerj.10691/supp-2 Supplemental Information 2 Raw data

10.7717/peerj.10691/supp-3 Supplemental Information 3 Clinical characteristics of patients with T2DM between NAFLD group and non-NAFLD group SUA, serum uric acid; BMI, body mass index; SBP, systolic blood pressure; DBP, diastolic blood pressure; BUN, blood urea nitrogen; Scr, serum creatinine; eGFR, estimated glomerular filtration rate; ALB, urinary microalbumin; TC, total cholesterol; TG, triglycerides, HDL-c, high-density lipoprotein-cholesterol; LDL, low-density lipoprotein-cholesterol; FFA, free fatty acid; FPG, fasting plasma glucose; 2h-PG, 2 h postprandial plasma glucose; HbA1c%, glycosylated hemoglobin Data are expressed as mean ± SD, number (percentage), and median (interquartile ranges). *Represented that the difference was significant.

10.7717/peerj.10691/supp-4 Supplemental Information 4 Clinical characteristics of patients with T2DM between DN group and non-DN group SUA, serum uric acid; BMI, body mass index; SBP, systolic blood pressure; DBP, diastolic blood pressure; BUN, blood urea nitrogen; Scr, serum creatinine; eGFR, estimated glomerular filtration rate; ALB, urinary microalbumin; TC, total cholesterol; TG, triglycerides, HDL-c, high-density lipoprotein-cholesterol; LDL, low-density lipoprotein-cholesterol; FFA, free fatty acid; FPG, fasting plasma glucose; 2h-PG, 2 h postprandial plasma glucose; HbA1c%, glycosylated hemoglobin Data are expressed as mean ± SD, number (percentage), and median (interquartile ranges). *Represented that the difference was significant.

10.7717/peerj.10691/supp-5 Supplemental Information 5 Clinical characteristics of patients with T2DM between DR group and non-DR group SUA, serum uric acid; BMI, body mass index; SBP, systolic blood pressure; DBP, diastolic blood pressure; BUN, blood urea nitrogen; Scr, serum creatinine; eGFR, estimated glomerular filtration rate; ALB, urinary microalbumin; TC, total cholesterol; TG, triglycerides, HDL-c, high-density lipoprotein-cholesterol; LDL, low-density lipoprotein-cholesterol; FFA, free fatty acid; FPG, fasting plasma glucose; 2h-PG, 2 h postprandial plasma glucose; HbA1c%, glycosylated hemoglobin Data are expressed as mean ± SD, number (percentage), and median (interquartile ranges). *Represented that the difference was significant.

10.7717/peerj.10691/supp-6 Supplemental Information 6 Clinical characteristics of patients with T2DM between DPN group and non-DPN group SUA, serum uric acid; BMI, body mass index; SBP, systolic blood pressure; DBP, diastolic blood pressure; BUN, blood urea nitrogen; Scr, serum creatinine; eGFR, estimated glomerular filtration rate; ALB, urinary microalbumin; TC, total cholesterol; TG, triglycerides, HDL-c, high-density lipoprotein-cholesterol; LDL, low-density lipoprotein-cholesterol; FFA, free fatty acid; FPG, fasting plasma glucose; 2h-PG, 2 h postprandial plasma glucose; HbA1c%, glycosylated hemoglobin Data are expressed as mean ± SD, number (percentage), and median (interquartile ranges). *Represented that the difference was significant.

## Additional Information and Declarations

### Data Availability

The following information was supplied regarding data availability: The raw measurements are available in the Supplementary Files.
