# Supplementary material for: The association between serum uric acid and diabetic complications in patients with type 2 diabetes mellitus by gender: a cross-sectional study
Source: PeerJ. 2021 Jan 13;9:e10691. doi: 10.7717/peerj.10691 (PMC7811288; doi:10.7717/peerj.10691)
Supplement: Supplemental Information 3 — SUA, serum uric acid; BMI, body mass index; SBP, systolic blood pressure; DBP, diastolic blood pressure; BUN, blood urea nitrogen; Scr, serum creatinine; eGFR, estimated glomerular filtration rate; ALB, urinary microalbumin; TC, total cholesterol; TG, triglycerides, HDL-c, high-density lipoprotein-cholesterol; LDL, low-density lipoprotein-cholesterol; FFA, free fatty acid; FPG, fasting plasma glucose; 2h-PG, 2 h postprandial plasma glucose; HbA1c%, glycosylated hemoglobin Data are expressed as mean ± SD, number (percentage), and median (interquartile ranges). *Represented that the difference was significant. [file peerj-09-10691-s003.docx]

**Table.2 Clinical characteristics of patients with T2DM between NAFLD group and non-NAFLD group**

|  | **Male** | | | **Female** | | |
| --- | --- | --- | --- | --- | --- | --- |
|  | NAFLD | non-NAFLD | P value | NAFLD | non-NAFLD | P value |
| N(%) | 774(43.4) | 1010(56.6) |  | 452(44.1) | 573(55.9) |  |
| age (year) | 52.4±11.7 | 55.5±12.2 | <0.001* | 59.3±10.9 | 60.6±11.7 | 0.057 |
| duration (years) | 4.0(1.0,9.0) | 5(1.0,10.0) | <0.001* | 5(1,10) | 7(2,12) | 0.002* |
| BMI（kg/m^2^) | 26.4±3.1 | 24.5±3.2 | <0.001* | 26.3±3.7 | 23.7±3.3 | <0.001* |
| SBP(mmHg) | 131.4±17.0 | 129.0±17.3 | 0.003* | 133.1±18.8 | 129.9±18.4 | 0.007* |
| DBP(mmHg) | 80.0±10.7 | 77.2±10.7 | <0.001* | 75.7±11.6 | 74.0±9.6 | 0.012* |
| BUN(mmol/L) | 5.5±1.4 | 5.8±1.8 | 0.102 | 5.2±1.8 | 5.4±1.8 | 0.217 |
| Scr(μmol/L) | 71.1(62.4,81.8) | 71.2(62.6,52.6) | 0.234 | 54.2(46.9,62.5) | 55.4(47.9,65.7) | 0.068 |
| eGFR（mL/min/1.73m^2^) | 108.0（93.4,120.0） | 106.5（88.7,117.4） | 0.005* | 98.5(89.0,108.6) | 96.5(85.5,106.4) | 0.01* |
| ALB(mg/24h) | 8.8（2.9,24.9） | 6.9（0,24.0） | 0.042* | 7.3（1.8,23.9） | 7.6（3.2,21.8） | 0.382 |
| UA(μmol/L) | 347.7±96.7 | 326.1±94.2 | <0.001* | 298.3±86.8 | 273.9±95.3 | <0.001* |
| TC(mmol/L) | 4.8±1.1 | 4.6±1.2 | 0.002* | 4.9±1.2 | 4.7±1.3 | 0.016* |
| TG(mmol/L) | 2.2（1.4,3.5） | 1.5（1.1,2.4） | <0.001* | 1.9(1.3,2.8) | 1.5(1.0,2.1) | <0.001* |
| HDL-c(mmol/L) | 0.97±0.26 | 1.01±0.28 | 0.007* | 1.08±0.26 | 1.15±0.34 | <0.001* |
| LDL-c(mmol/L) | 2.82±0.90 | 2.80±0.95 | 0.634 | 2.93±0.96 | 2.78±0.99 | 0.022* |
| FFA(μmol/L) | 486.7(366.1,626.6) | 461.9(327.7,601.0) | 0.077 | 521.8(360.8,682.9) | 489.6(316.1,649.5) | 0.184 |
| FBG(mmol/L) | 8.5(6.4,11.2) | 8.3(6.2,11.4) | 0.453 | 8.2(6.3,10.9) | 8.1(5.7,10.8) | 0.144 |
| 2h-PG(mmol/L) | 18.0±4.9 | 18.1±5.7 | 0.821 | 18.6±5.2 | 18.2±6.0 | 0.33 |
| HbA1c(%) | 8.6(7.1,10.2) | 8.3(6.9,10.3) | 0.192 | 8.4(7.3,10.0) | 8.0(6.8,10.2) | 0.085 |

SUA, serum uric acid; BMI, body mass index; SBP, systolic blood pressure; DBP, diastolic blood pressure; BUN, blood urea nitrogen; Scr, serum creatinine; eGFR, estimated glomerular filtration rate; ALB, urinary microalbumin; TC, total cholesterol; TG, triglycerides, HDL-c, high-density lipoprotein-cholesterol; LDL, low-density lipoprotein-cholesterol; FFA, free fatty acid; FPG, fasting plasma glucose; 2h-PG, 2h postprandial plasma glucose; HbA1c%, glycosylated hemoglobin

Data are expressed as mean ± SD, number (percentage), and median (interquartile ranges). *Represented that the difference was significant.
