# Supplementary material for: The association between serum uric acid and diabetic complications in patients with type 2 diabetes mellitus by gender: a cross-sectional study
Source: PeerJ. 2021 Jan 13;9:e10691. doi: 10.7717/peerj.10691 (PMC7811288; doi:10.7717/peerj.10691)
Supplement: Supplemental Information 4 — SUA, serum uric acid; BMI, body mass index; SBP, systolic blood pressure; DBP, diastolic blood pressure; BUN, blood urea nitrogen; Scr, serum creatinine; eGFR, estimated glomerular filtration rate; ALB, urinary microalbumin; TC, total cholesterol; TG, triglycerides, HDL-c, high-density lipoprotein-cholesterol; LDL, low-density lipoprotein-cholesterol; FFA, free fatty acid; FPG, fasting plasma glucose; 2h-PG, 2 h postprandial plasma glucose; HbA1c%, glycosylated hemoglobin Data are expressed as mean ± SD, number (percentage), and median (interquartile ranges). *Represented that the difference was significant. [file peerj-09-10691-s004.docx]

**Table.3. Clinical characteristics of patients with T2DM between DN group and non-DN group**

|  | **Male** | | | **Female** | | |
| --- | --- | --- | --- | --- | --- | --- |
|  | DN | non-DN | P value | DN | non-DN | P value |
| N | 292(16.7) | 1492(83.3) |  | 145(14.2) | 875(85.8) |  |
| age (year) | 59.4±12.4 | 53.1±11.7 | <0.001* | 64.3±9.8 | 59.4±11.4 | 0.001* |
| duration (years) | 7.0(2.0,10.0) | 4.0(1.0,10.0) | <0.001* | 8.0(2.0,12.0) | 6.0(1.0,10.0) | 0.033* |
| BMI（kg/m^2^) | 25.6±3.5 | 25.3±3.2 | 0.12 | 25.2±3.7 | 24.9±3.6 | 0.408 |
| SBP(mmHg) | 136.4±18.1 | 128.8±16.8 | 0.001* | 138.7±19.9 | 130.1±18.1 | <0.001* |
| DBP(mmHg) | 78.7±11.9 | 78.3±10.6 | 0.604 | 76.5±10.9 | 74.5±10.5 | 0.035* |
| BUN(mmol/L) | 6.5±2.4 | 5.5±1.4 | <0.001* | 6.5±3.1 | 5.1±1.4 | <0.001* |
| Scr(μmol/L) | 86.1(69.5,108.3) | 70.0(61.7,79.0) | <0.001* | 66.9（54.1,94.8） | 54.0（46.8,62.0） | <0.001* |
| eGFR（mL/min/1.73m^2^) | 85.8(58.6,110.2) | 109.0(95.5,119.6) | <0.001* | 81.8(52.0,97.8) | 98.7(89.3,108.6) | <0.001* |
| ALB(mg/24h) | 53.2(36.5,73.9) | 5.0(0,11.3) | <0.001* | 50.0(35.8,68.7) | 5.2(0,11.2) | <0.001* |
| UA(μmol/L) | 350.6±96.9 | 332.5±95.4 | 0.003* | 320.1±114.2 | 278.9±87.0 | <0.001* |
| TC(mmol/L) | 4.8±1.3 | 4.6±1.2 | 0.077 | 4.9±1.1 | 4.7±1.3 | 0.185 |
| TG(mmol/L) | 2.0(1.3,3.5) | 1.7(1.2,2.8) | 0.002* | 1.8(1.3,2.9) | 1.6(1.2,2.4) | 0.002* |
| HDL-c(mmol/L) | 0.97±0.24 | 1.00±0.28 | 0.09 | 1.09±0.23 | 1.12±0.32 | 0.196 |
| LDL-c(mmol/L) | 2.84±1.00 | 2.80±0.91 | 0.486 | 2.87±0.88 | 2.84±1.00 | 0.77 |
| FFA(μmol/L) | 419.1(276.5,523.7) | 476.3(348.3,613.4) | 0.124 | 582.1(482.8,748.5) | 504.8(336.7,651.7) | 0.115 |
| FBG(mmol/L) | 7.7(5.8,10.6) | 8.5(6.4,11.5) | 0.008* | 7.8(5.5,9.9) | 8.2(6.1,11.0) | 0.045* |
| 2h-PG(mmol/L) | 18.0±5.4 | 18.1±5.4 | 0.903 | 18.4±5.3 | 18.4±5.7 | 0.928 |
| HbA1c(%) | 8.1(7.1,10.0) | 8.5(6.9,10.3) | 0.583 | 8.1(6.9,9.8) | 8.3(7.0,10.1) | 0.701 |

SUA, serum uric acid; BMI, body mass index; SBP, systolic blood pressure; DBP, diastolic blood pressure; BUN, blood urea nitrogen; Scr, serum creatinine; eGFR, estimated glomerular filtration rate; ALB, urinary microalbumin; TC, total cholesterol; TG, triglycerides, HDL-c, high-density lipoprotein-cholesterol; LDL, low-density lipoprotein-cholesterol; FFA, free fatty acid; FPG, fasting plasma glucose; 2h-PG, 2h postprandial plasma glucose; HbA1c%, glycosylated hemoglobin

Data are expressed as mean ± SD, number (percentage), and median (interquartile ranges). *Represented that the difference was significant.
