# Supplementary material for: The association between serum uric acid and diabetic complications in patients with type 2 diabetes mellitus by gender: a cross-sectional study
Source: PeerJ. 2021 Jan 13;9:e10691. doi: 10.7717/peerj.10691 (PMC7811288; doi:10.7717/peerj.10691)
Supplement: Supplemental Information 5 — SUA, serum uric acid; BMI, body mass index; SBP, systolic blood pressure; DBP, diastolic blood pressure; BUN, blood urea nitrogen; Scr, serum creatinine; eGFR, estimated glomerular filtration rate; ALB, urinary microalbumin; TC, total cholesterol; TG, triglycerides, HDL-c, high-density lipoprotein-cholesterol; LDL, low-density lipoprotein-cholesterol; FFA, free fatty acid; FPG, fasting plasma glucose; 2h-PG, 2 h postprandial plasma glucose; HbA1c%, glycosylated hemoglobin Data are expressed as mean ± SD, number (percentage), and median (interquartile ranges). *Represented that the difference was significant. [file peerj-09-10691-s005.docx]

**Table.4. Clinical characteristics of patients with T2DM between DR group and non-DR group**

|  | **Male** | | | **Female** | | |
| --- | --- | --- | --- | --- | --- | --- |
|  | DR | non-DR | P value | DR | non-DR | P value |
| N(%) | 141(8.1) | 1642(91.9) |  | 99(9.7) | 921(90.3) |  |
| age (year) | 56.5±9.9 | 54.0±12.2 | 0.006* | 62.1±9.4 | 59.8±11.5 | 0.03* |
| duration (years) | 8.0（4.0,12.0） | 4.5(1.0,10.0） | <0.001* | 10.0(4.0,13.0) | 6.0(1.0,10.0) | 0.001* |
| BMI（kg/m^2^) | 25.7±3.3 | 25.3±3.3 | 0.151 | 25.0±3.6 | 24.9±3.6 | 0.887 |
| SBP(mmHg) | 133.4±20.1 | 129.8±16.9 | 0.039* | 134.9±17.6 | 130.9±18.7 | 0.043* |
| DBP(mmHg) | 78.4±11.9 | 78.4±10.7 | 0.977 | 74.4±9.8 | 74.8±10.7 | 0.704 |
| BUN(mmol/L) | 6.0±2.4 | 5.6±1.6 | 0.089 | 5.6±1.5 | 5.3±1.8 | 0.121 |
| Scr(μmol/L) | 74.0(63.1,86.5) | 71.0(62.4,81.9) | 0.033* | 56.0(49.0,67.2) | 54.9(47.0,63.6) | 0.057 |
| eGFR（mL/min/1.73m^2^) | 102.9(83.5,114.3) | 107.8(92.0,118.9) | 0.001* | 95.6(79.9,102.1) | 97.8(87.7,107.8) | 0.018* |
| ALB(mg/24h) | 11.7(2.9,40.0) | 7.5(0,23.8) | 0.017* | 8.7(3.9,31.2) | 7.1(2.5,20.8) | 0.136 |
| UA(μmol/L) | 331±92.9 | 335.9±96.2 | 0.562 | 287.9±98.6 | 284.4±91.8 | 0.72 |
| TC(mmol/L) | 4.5±1.2 | 4.7±1.2 | 0.086 | 4.7±1.1 | 4.8±1.2 | 0.803 |
| TG(mmol/L) | 1.6(1.1,2.6) | 1.8(1.2,2.9) | 0.025* | 1.6(1.2,2.4) | 1.7(1.2,2.4) | 0.915 |
| HDL-c(mmol/L) | 1.01±0.31 | 0.99±0.27 | 0.454 | 1.12±0.24 | 1.12±0.32 | 0.956 |
| LDL-c(mmol/L) | 2.67±0.92 | 2.82±0.93 | 0.08 | 2.82±0.92 | 2.85±0.99 | 0.741 |
| FFA(μmol/L) | 407.8(312.4,520.4) | 480.7(350.3,618.8) | 0.022* | 594.7(366.1,792.5) | 498.5(333.7,649.5) | 0.128 |
| FBG(mmol/L) | 8.8(6.5,12.1) | 8.4(6.2,11.3) | 0.23 | 8.5(6.3,11.7) | 8.0(6.0,10.8) | 0.267 |
| PBG(mmol/L) | 18.1±5.1 | 18.1±5.4 | 0.936 | 19.2±5.5 | 18.3±5.7 | 0.113 |
| HbA1c(%) | 8.0(7.0,9.8) | 8.5(7.0,10.3) | 0.316 | 8.3(7.3,10.2) | 8.3(6.9,10.1) | 0.252 |

SUA, serum uric acid; BMI, body mass index; SBP, systolic blood pressure; DBP, diastolic blood pressure; BUN, blood urea nitrogen; Scr, serum creatinine; eGFR, estimated glomerular filtration rate; ALB, urinary microalbumin; TC, total cholesterol; TG, triglycerides, HDL-c, high-density lipoprotein-cholesterol; LDL, low-density lipoprotein-cholesterol; FFA, free fatty acid; FPG, fasting plasma glucose; 2h-PG, 2h postprandial plasma glucose; HbA1c%, glycosylated hemoglobin

Data are expressed as mean ± SD, number (percentage), and median (interquartile ranges). *Represented that the difference was significant.
