# Supplementary material for: The association between serum uric acid and diabetic complications in patients with type 2 diabetes mellitus by gender: a cross-sectional study
Source: PeerJ. 2021 Jan 13;9:e10691. doi: 10.7717/peerj.10691 (PMC7811288; doi:10.7717/peerj.10691)
Supplement: Supplemental Information 6 — SUA, serum uric acid; BMI, body mass index; SBP, systolic blood pressure; DBP, diastolic blood pressure; BUN, blood urea nitrogen; Scr, serum creatinine; eGFR, estimated glomerular filtration rate; ALB, urinary microalbumin; TC, total cholesterol; TG, triglycerides, HDL-c, high-density lipoprotein-cholesterol; LDL, low-density lipoprotein-cholesterol; FFA, free fatty acid; FPG, fasting plasma glucose; 2h-PG, 2 h postprandial plasma glucose; HbA1c%, glycosylated hemoglobin Data are expressed as mean ± SD, number (percentage), and median (interquartile ranges). *Represented that the difference was significant. [file peerj-09-10691-s006.docx]

**Table.5. Clinical characteristics of patients with T2DM between DPN group and non-DPN group**

|  | **Male** | | | **Female** | | |
| --- | --- | --- | --- | --- | --- | --- |
|  | DPN | non-DPN | P value | DPN | non-DPN | P value |
| N | 207(11.8) | 1577(88.2) |  | 121(6.9) | 899 (93.4) |  |
| age (year) | 59.0±10.4 | 53.5±12.1 | <0.001* | 63.7±10.2 | 59.6±11.4 | <0.001* |
| duration (years) | 8.0(3.0,13.0) | 4（1,10） | <0.001* | 10.0（5.0,16.0） | 6.0（1.0,10.0） | <0.001* |
| BMI（kg/m^2^) | 24.9±3.3 | 25.4±3.3 | 0.056 | 25.4±3.8 | 24.9±3.6 | 0.11 |
| SBP(mmHg) | 131.7±18.0 | 129.8±17.1 | 0.134 | 135.2±20.2 | 130.8±18.4 | 0.015* |
| DBP(mmHg) | 77.2±10.7 | 78.6±10.8 | 0.089 | 74.3±10.2 | 74.8±18.4 | 0.612 |
| BUN(mmol/L) | 6.0±1.7 | 5.6±1.6 | 0.003* | 5.5±1.7 | 5.3±1.8 | 0.14 |
| Scr(μmol/L) | 73.0(63.9,85.3) | 71.0(62.4,81.9) | 0.054 | 56.2(48.8,65.0) | 54.9(47.0,63.9) | 0.107 |
| eGFR（mL/min/1.73m^2^) | 99.4(84.8,113.6) | 108.1(91.9,119.5) | <0.001* | 92.6(81.9,103.5) | 97.9(87.9,107.8) | 0.008* |
| ALB(mg/24h) | 10.5(0,28.1) | 7.7(0,23.8) | 0.307 | 8.6(3.6,26.7) | 7.1(2.5,22.9) | 0.291 |
| UA(μmol/L) | 334.6±88.4 | 335.6±96.8 | 0.892 | 285.8±96.4 | 284.6±91.9 | 0.891 |
| TC(mmol/L) | 4.5±1.1 | 4.7±1.2 | 0.017* | 4.7±1.1 | 4.8±1.2 | 0.483 |
| TG(mmol/L) | 1.5(1.0,2.4) | 1.8(1.2,3.0) | <0.001* | 1.6(1.1,2.2) | 1.7(1.2,2.5) | 0.303 |
| HDL-c(mmol/L) | 1.06±0.30 | 0.98±0.27 | <0.001* | 1.16±0.32 | 1.11±0.31 | 0.114 |
| LDL-c(mmol/L) | 2.64±0.88 | 2.83±0.93 | 0.006* | 2.76±0.92 | 2.86±0.99 | 0.295 |
| FFA(μmol/L) | 423.8(312.8,580.7) | 479.2(351.8,622.8) | 0.094 | 492.0(329.9,631.9) | 512.6(343.8,657.5) | 0.525 |
| FBG(mmol/L) | 8.5（6.1,12.5） | 8.4（6.3,11.1) | 0.336 | 7.9(6.1,11.0) | 8.2(6.0,10.9) | 0.926 |
| 2h-PG(mmol/L) | 18.4±5.6 | 18.0±5.4 | 0.323 | 18.7±5.3 | 18.3±5.7 | 0.473 |
| HbA1c(%) | 8.0(6.8,9.9) | 8.5(7.0,10.4) | 0.215 | 7.8(7.0,9.8) | 8.4((7.0,10.1) | 0.154 |

SUA, serum uric acid; BMI, body mass index; SBP, systolic blood pressure; DBP, diastolic blood pressure; BUN, blood urea nitrogen; Scr, serum creatinine; eGFR, estimated glomerular filtration rate; ALB, urinary microalbumin; TC, total cholesterol; TG, triglycerides, HDL-c, high-density lipoprotein-cholesterol; LDL, low-density lipoprotein-cholesterol; FFA, free fatty acid; FPG, fasting plasma glucose; 2h-PG, 2h postprandial plasma glucose; HbA1c%, glycosylated hemoglobin

Data are expressed as mean ± SD, number (percentage), and median (interquartile ranges). *Represented that the difference was significant.
